# Longitudinal Evaluation of Pain, Flare‐Up, and Emotional Health in Fibrodysplasia Ossificans Progressiva: Analyses of the International FOP Registry

**DOI:** 10.1002/jbm4.10181

**Published:** 2019-03-01

**Authors:** Ke Peng, Kin Cheung, Arielle Lee, Christine Sieberg, David Borsook, Jaymin Upadhyay

**Affiliations:** ^1^ Center for Pain and the Brain Department of Anesthesiology Critical Care and Pain Medicine Boston Children's Hospital Harvard Medical School Boston MA USA; ^2^ BioSAS Consulting, Inc. Wellesley MA USA; ^3^ Biobehavioral Pediatric Pain Laboratory Department of Psychiatry Boston Children's Hospital Boston MA USA

**Keywords:** FIBRODYSPLASIA OSSIFICANS PROGRESSIVA, FLARE‐UP, PAIN, EMOTIONAL HEALTH, PHYSICAL HEALTH

## Abstract

Fibrodysplasia ossificans progressiva (FOP) is an ultra‐rare, inherited, connective tissue disease with ∼800 documented cases worldwide. The principal pathological feature of FOP is the transition of skeletal muscle, tendons, ligaments, and fascia into cartilage and bone. This heterotopic ossification (HO) is often preceded by painful soft tissue swellings or flare‐ups that may last several months. For many individuals, experiencing a flare‐up may represent a worsening of their condition and contribute to feelings of anxiety or suppressed affect, both of which are well‐recognized to exacerbate pain perception. To date, much remains unknown regarding the dynamics of pain and emotional health in FOP during flare‐up and also quiescent, non–flare‐up disease phases. In order to elucidate the occurrence and effect of pain in FOP, this study analyzed Patient‐Reported Outcomes Measurement Information System–based questionnaires completed by 99 patients participating in the international FOP Registry over a 30‐month period. We observed that although moderate to severe pain (≥4, 0 to 10 pain scale) was commonly associated with flare‐ups (56% to 67%), surprisingly, 30% to 55% of patients experienced similar pain levels during non–flare‐up states. In those patients reporting pain levels of ≥4, 45% to 74% of patients report experiencing anxiety, depression, or irritability, with 36% to 48% reporting emotional problems during no to mild pain states. Furthermore, independent of the flare‐up status, the severity of pain in FOP patients was found to be significantly anti‐correlated with emotional health, physical health, and overall quality‐of‐life. These findings strongly suggest the need for an improved understanding of pain and emotional health in FOP during flare‐up and quiescent periods. © 2019 The Authors. *JBMR Plus* published by Wiley Periodicals, Inc. on behalf of American Society for Bone and Mineral Research.

## Introduction

Fibrodysplasia ossificans progressiva (FOP) is an ultra‐rare (1:1,400,000 prevalence worldwide) and disabling inherited disorder that arises from missense mutations of the type I bone morphogenetic protein (BMP) receptor Activin A receptor type 1 (ACVR1).^1^, ^2^ To date, approximately 800 FOP cases have been documented. FOP is principally characterized by skeletal dysplasia and episodic heterotopic ossification (HO) of skeletal muscles, tendons, ligaments, and fascia.^3^ Clinical observations have shown that induction or growth of HO lesions is often preceded by severely painful edematous soft tissue swellings termed “flare‐ups.”^4^, ^5^, ^6^ Flare‐ups may occur in regions of the hips, knees, back, neck, head, and shoulders and can last several weeks with a concomitant presence of fever, inflammation, and loss of mobility.^7^, ^8^, ^9^ In addition to yielding high‐levels of pain and physical debilitation, these noxious events can elicit depression or feelings of anxiety, which can in turn heighten pain perception.

Flare‐ups and injury to the musculoskeletal system are undoubtedly principal sources of pain in FOP, yet past reports have noted the presence of recurrent severe headache (migraine, cluster headaches, tension‐type headache), neuropathic pain of the lower extremities, and somatosensory abnormalities (eg, allodynia, hyperalgesia, numbness, and tingling).^5^, ^10^ This heterogeneity in pain phenotypes may in part underlie why pain management across FOP patients currently remains a difficult process. Relatedly, much remains unknown in FOP with regard to pain trajectories as well as emotional and physical health or the dynamics of these interrelated entities during flare‐up or clinically quiescent, non–flare‐up periods. In order to facilitate a better understanding among flare‐ups, pain, mental health, and physical health, we performed an analysis of the FOP patient registry, which was created and is managed by the International Fibrodysplasia Ossificans Progressiva Association (IFOPA).^11^ This ongoing, worldwide patient registry consists of self‐reported data including baseline demographics, disease activity measures (ie, occurrence of flare‐ups and HO), pain, and health status over a 30‐month period in adolescent and adult FOP patients. Across the 30‐month period, patients participating in the registry completed clinical questionnaires derived from the Patient‐Reported Outcomes Measurement Information System (PROMIS).^12^

In the current study, we aimed to test the hypothesis that although high levels of pain may be periodically driven by a flare‐up event, perception of pain along with a patient's emotional health are altered during non–flare‐up states. Furthermore, we explored how emotional and physical health as well as quality‐of‐life in FOP patients varied as a function of pain severity. Understanding pain in patients diagnosed with FOP will assist in defining an initial paradigm that may enhance the evaluation of these patients in the clinic and may provide an important first step toward the use of objective measures of pain in this patient population.

## Materials and Methods

### The FOP patient data registry

This investigation utilized patient reported data from the FOP Connection Registry. This international registry, spanning 42 counties, was initiated in July 2015 and has aimed to capture FOP patient demographics and disease information on a voluntary basis. The core objectives of this global initiative are to (i) organize the FOP community for potential participation in clinical investigations, (ii) cogitate patient experiences, (iii) inform on treatment outcomes, and (iv) provide a more detailed understanding of the emotional, cognitive, and physical health in FOP patients over time.

Participation in the FOP registry was open to all adolescent and adult patients diagnosed with the disease by a physician or confirmed with genetic testing. No exclusion criteria were utilized. Each patient (or patient's parent or legal guardian) provided informed consent prior to participation into the registry. Approval of the patient portal, protocol, and informed consent form was obtained from the Chesapeake/Advarra Institutional Review Board (Columbia, MD, USA). Ethics approval specific to the current study was also obtained from the Boston Children's Hospital Institutional Review Board.

### Scope of patient data registry analyses

The FOP patient cohort currently analyzed does not include all individuals within the registry, but rather those adult patients (≥18 years of age) who were able to provide information on demographics, disease‐specific information (ie, age at FOP diagnosis and symptom onset) and flare‐up status as well as those individuals who completed the Patient‐Reported Outcomes Measurement Information System (PROMIS) database questionnaires relevant to the focus of this investigation. We also included only FOP patients who remained enrolled in the registry across a 30‐month period (between July 1, 2015, and September 20, 2018) who provided data through the patient portal at enrollment, 6‐month, 12‐month, 18‐month, 24‐month, and 30‐month time points.

### Study measures

In addition to flare‐up status, multiple metrics from the PROMIS‐based questionnaires and other sources were assessed. Between enrollment and 30 months, patients completed the following questions, where responses were subdivided into two categories:

- Pain: “How would you rate your pain on average?”
  Scale: 0 = no pain; 10 = worst imaginable pain
  Category 1: No to mild pain: 0 to 3
  Category 2: Moderate to severe pain: ≥4
- Emotional health: “How often have you been bothered by emotional problems such as feeling anxious, depressed or irritable?”
  Scale: Never, rarely, sometimes, often, or always
  Category 1: Never and rarely (without Emotional Problems)
  Category 2: Sometimes, often or always (with Emotional Problems)
- Mental health: “In general, how would you rate your mental health, including your mood and your ability to think?”
  Scale: Excellent, very good, good, fair, or poor
  Category 1: Excellent, very good, and good (+ Mental Health)
  Category 2: Fair or poor (− Mental Health)
- Physical health: “In general, how would you rate your physical health?”
  Scale: Excellent, very good, good, fair, or poor
  Category 1: Excellent, very good, and good (+ Physical Health)
  Category 2: Fair or poor (− Physical Health)
- Quality‐of‐life: “In general, what would you say your quality‐of‐life is?”
  Scale: Excellent, very good, good, fair, or poor
  Category 1: Excellent, very good, and good (+ Quality of Life)
  Category 2: Fair or poor (− Quality of Life)
- Sleep (Non‐PROMIS based): “Do you experience poor sleep quality, take a long time to fall asleep, snore, have excessive leg movements during sleep, or wake up with shortness of breath or difficulty breathing?”
  Scale: Yes, no, or unsure
- Neuropathic pain (Non‐PROMIS based): “Have you experienced new or continuing health issues related to the brain and nerves symptoms?” (brief description of neuropathic pain given).
  Scale: Yes, no, or unsure

### Statistical analyses

Summaries for continuous variables were reported with the number of observations (*n*), arithmetic mean, standard deviation, median, minimum, and maximum. Categorical variables were tabulated with frequencies and percentages. Statistical comparisons between two independent binomial proportions were performed using Fisher's exact test, whereas Spearman's rank correlation coefficients were calculated to explore the association between the cohorts or health‐related measure (ie, pain magnitude versus emotional health scores). Statistical significance was assessed at the 5% level.

## Results

### Baseline demographics

A total of 99 patients diagnosed with FOP were evaluated in the current investigation (Table 1). The majority of enrolled adult patients (34.5 ± 12.31 years of age) experienced FOP‐related symptoms and were diagnosed during early childhood years. Seventy percent of patient population was female and primarily reside in North American and European countries. The genetic background was known in 50% of patients.

**Table 1:** Baseline Demographics

Characteristic	Age ≥18 years (*n* = 99)
Enrollment age (years)	
*n*	99
Mean ± SD	34.8 ± 11.98
Median (minimum, maximum)	32 (18.0, 74.0)
Age at first symptom onset (years)	
*n*	98
Mean ± SD	6.9 ± 6.94
Median (minimum, maximum)	5.0 (0.1, 45.0)
Age at diagnosis (years)	
*n*	98
Mean ± SD	9.4 ± 7.93
Median (minimum, maximum)	8.0 (0.1, 48.0)
Gender, *n* (%)	
Male	30 (30.3)
Female	69 (69.7)
Race/ethnicity, *n* (%)	
Asian	6 (6.1)
White	79 (79.8)
Black or African American	1 (1.0)
American Indian or Alaska Native	1 (1.0)
Other/unknown/refuse to answer	9 (9.1)
Missing	3 (3.0)
Continent, *n* (%)	
Asia	5 (5.9)
Africa	2 (2.0)
Europe	32 (32.3)
Australia	6 (6.1)
North America	38 (38.4)
South America	16 (16.2)
Asia	5 (5.9)
Type of FOP, *n* (%)	
FOP variant	6 (6.1)
FOP classic (R206H mutation)	43 (43.4)
FOP, type not known/not sure	48 (48.5)
Missing	2 (2.0)

### Pain during flare‐up and non–flare‐up states

**Figure 1:**
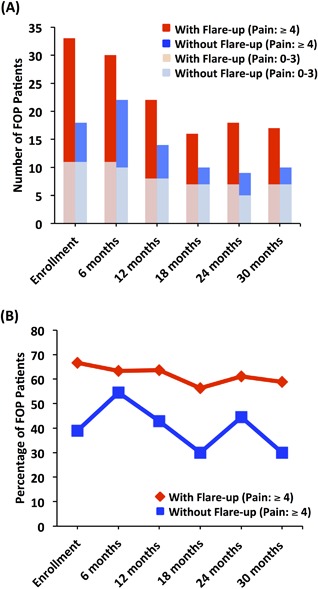
Pain in FOP during flare‐up and quiescent periods. (*A*) The number of patients with flare‐up and moderate to severe pain was greater compared to those patients reporting no flare‐up + moderate to severe pain. Across time points, a similar number of patients reported no to mild pain in either flare‐up or quiescent periods. Fisher's exact test *p* values: Enrollment = 0.078, 6‐month = 0.58, 12‐month = 0.31, 18‐month = 0.25, 24‐month = 0.45, and 30‐month = 0.24. (*B*) The percentage of patients reporting pain at a level ≥4 was consistently higher during flare‐up, but this percentage was considerable in the non–flare‐up state. See also Supplemental Fig. 1A.

In order to elucidate the relationship between flare‐up status and the reported pain severity, FOP patients were subdivided at each time point into patients with and without flare‐up as well as those who experienced no to mild (0 to 3 rating) and moderate to severe (4 to 10 rating) pain levels (Fig. 1). A very similar number of patients with and without flare‐up reported no to mild pain levels, with 33% to 44% of patients with flare‐up experiencing low levels of pain (Fig. 1 *A*, Supplemental Fig. 1A). Moderate to severe pain levels were in fact frequently associated with the occurrence of flare‐up; however, it is important to note that as high as 55% of patients (*n* = 12, 6 months post‐enrollment) who did not experience a flare‐up reported pain ≥4 (Fig. 1 *B*). Furthermore, from the pool of 99 FOP patients, 13% of individuals at enrollment noted neuropathic pain.

More incidents of fair to poor mental health (ie, ability to think) occurred in FOP patients with flare‐up, but overall a low percentage of patients reported mental health issues in either flare‐up condition (Supplemental Table 1). A higher percentage of patients reported emotional problems in the flare‐up group at each time point; however, cases of anxiety, depression, or irritability were common in the non–flare‐up condition as well, particularly at enrollment and 6‐month time points (Supplemental Table 2).

### Pain and mental and physical health status

**Figure 2:**
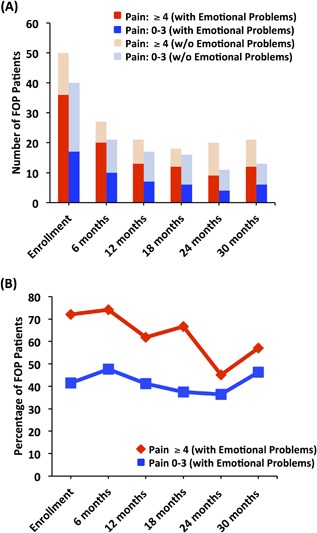
Emotional health during low and high pain states. (*A*) A substantial number of FOP patients reported moderate to severe pain along with emotional problems (ie, experiencing feelings of anxiety, depression, or irritability) compared to the no to mild pain cohort. The majority of patients across time reporting no to mild pain presented without emotional problems. Fisher's exact test *p* values: Enrollment = 0.0056, 6‐month = 0.077, 12‐month = 0.33, 18‐month = 0.17, 24‐month = 0.72, and 30‐month = 0.73. (*B*) The percentage of patients reporting emotional problems at a level ≥4 was consistently higher compared to patients reporting pain at a 0 to 3 level. See also Supplemental Fig. 1B.

**Figure 3:**
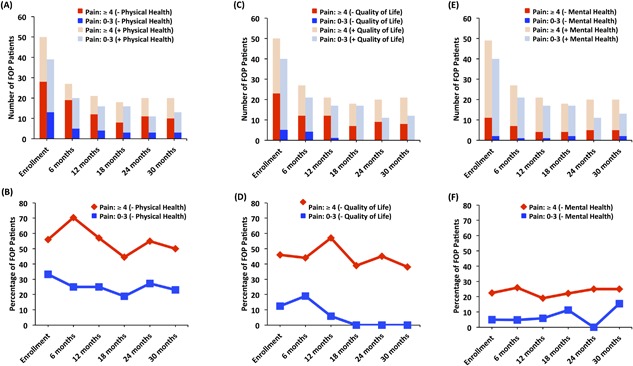
Impact of pain on physical health, mental health, and quality of life. (*A*, *B*) FOP patients reporting pain in the moderate to severe range more frequently reported fair to poor physical health. Fisher's exact test *p* values: Enrollment = 0.053, 6‐month = 0.0032, 12‐month = 0.093, 18‐month = 0.15, 24‐month = 0.26, and 30‐month = 0.16. (*C*, *D*) Mental health was impacted by moderate to severe pain levels, but the impact of pain on mental health was primarily noted at the enrollment time point. Fisher's exact test *p* values: Enrollment = 0.032, 6‐month = 0.064, 12‐month = 0.35, 18‐month = 0.66, 24‐month = 0.13, and 30‐month = 0.68. (*E*, *F*) Across the majority of evaluation time points, FOP patients with moderate to severe pain demonstrated a concomitant level of fair to poor quality of life. Fisher's exact test *p* values: Enrollment = 0.006, 6‐month = 0.075, 12‐month = 0.0015, 18‐month = 0.0076, 24‐month = 0.012, and 30‐month = 0.03. See also Supplemental Fig. 1C–E.

FOP patients experiencing moderate to severe pain more frequently reported emotional problems. However, experiencing anxiety, depression, or irritability was a substantial issue in the no to mild pain cohort (Fig. 2 *A*). Across the 30‐month evaluation period, upward of 74% and 48% of patients reported experiencing emotional deficits in the moderate to severe and no to mild pain level subpopulations, respectively (Fig. 2 *B*). Likewise, FOP patients living with pain also consistently reported a higher degree of fair to poor physical health (Fig. 3 *A*, *B*) and quality of life (Fig. 3 *C*, *D*). Issues related to mental health were not frequent in either low or high pain states (Fig. 3 *E*, *F*), indicating generally intact cognitive capabilities at the group‐level and over time. In Supplemental Fig. 1B‐E, the percentage of FOP patients in either pain state and who did not report emotional, mental, or physical health problems are depicted.

In addition to the focus on flare‐up and pain, the association between psychosocial functioning and other health‐related parameters were evaluated. It is noted that apart from the enrollment time point, experiencing fair to poor emotional health did not lead to substantially more FOP patients reporting lower than normal physical health or poor quality of life (Supplemental Tables 3 and 4). As expected, a higher percentage of patients reporting fair to poor quality of sleep did experience emotional and cognitive health problems, yet individuals without sleep problems experienced similar emotional health issues (Supplemental Tables 5 and 6). For example, at enrollment and 6‐month follow‐up, 51% (*n* = 28) and 55% (*n* = 18) of FOP patient experiencing no sleep problems reported feeling anxiety, depressed, or irritable.

### Correlation between pain and other health‐related measures

In order to further determine the association between pain severity in FOP and measures informing on patient psychological and physical status, Spearman correlation analyses were performed using all data points collected at enrollment (Table 2). Robust and significant anti‐correlations were observed between patient‐reported pain severity and emotional health, mental health, physical health, and overall quality‐of‐life. Specifically, across ∼90 FOP patients, higher pain levels corresponded to diminished patient well‐being as defined by multiple health‐related metrics.

**Table 2:** Association of Pain and Other Health‐Related Measures

Characteristics	*n*	Spearman coefficient	Spearman *p*
Pain versus quality of life	90	–0.5	<0.0001
Pain versus physical health	89	–0.4	0.0001
Pain versus mental health	89	–0.39	0.0001
Pain versus emotional health	90	–0.36	0.0005

## Discussion

This investigation importantly shows the commonality and impact of pain in FOP under flare‐up and non–flare‐up states. It is often considered that pain in FOP is a symptom that primarily arises during flare‐ups and perhaps HO growth.^5^ Certainly, physical changes that occur during a flare‐up episode, eg, increased and sustained pressure on hard or soft tissue structures of the musculoskeletal and nervous systems, can induce an increase in afferent signaling within peripheral and central pain pathways. Such pathological episodes may at times involve severe levels of pain and can also be emotionally taxing and physically debilitating; two experiences that can magnify the level of pain perceived. A key finding stemming from the current work, however, is that moderate to severe pain is present in individuals during non–flare‐up or what are thought to be quiescent periods. This observation suggests the presence of a chronic pain background in subpopulations of FOP patients, and also, a discordance between peripheral pathological mechanisms (ie, inflammation, edema, or HO growth) and the magnitude of perceived pain. An important component of future work should be to decipher if regions of the body presenting with pain, yet are considered subclinical in terms of detectable flare‐up, relate to induction of new HO lesions or expansion of existing HO. Moreover, in other conditions involving the musculoskeletal system such as fibrous dysplasia^13^ or juvenile idiopathic arthritis,^14^ it is also often the case where patient‐reported pain is not commensurate with detectable peripheral pathology. As such, there are perhaps overlapping mechanisms at play that drive the pain‐peripheral pathology disconnect commonly observed in musculoskeletal diseases, and by comprehending underlying pain processes in one condition, insights into causes of pain may be gained in other clinical cases.

Equally critical, FOP patients reporting moderate to severe pain, whether under flare‐up or non–flare‐up conditions, were associated with lower levels of emotional and physical health as well as poorer quality of life compared to individuals reporting no to mild levels of pain. Although pain may indeed elicit aberrancies in mental and physical health and lower the overall quality of life,^15^, ^16^ the latter components may in part be attributed to having a chronic and serious life‐limiting illness. This can differ from other more common pain conditions (eg, acute postsurgical pain), in which many facets of a patient's daily activity are kept intact over a long‐term basis.

The current analysis of the FOP registry^11^ focused on patients who were 18 years of age or more at enrollment and who completed questionnaires including those derived from the PROMIS database.^12^ In these adult patients, although factors such as pain or diminished emotional health and quality of life may have resulted from disease processes and activity (ie, flare‐ups, HO expansion, and loss of physical mobility) occurring during adulthood, past pathological events and FOP‐related symptoms experienced in childhood to late juvenile states are important to consider. For many FOP patients, symptoms of the disease as well as HO induction and expansion can take place as early as infancy. (In the cohort analyzed herein, FOP related symptoms were first noted at 6.9 ± 6.94 years of age and the age at enrollment was 34.8 ± 11.98 years [Table 1].) A variable source of pain and dysfunction in adult FOP patients that must specifically be considered is the early presence of degenerative joint disease and joint dysfunction caused by widespread and severe skeletal dysplasia. Therefore, a pain phenotype and, relatedly, an increased propensity toward experiencing psychological symptoms in later years of a FOP patient's lifetime may in part be a downstream effect of enhanced pain, physical dysfunction, and psychological stress levels initiated during early stages.^17^, ^18^ Independent of the clinical condition, providing effective pain and psychological treatment to childhood or juvenile patients can be a challenging process. Many young individuals have difficulty in communicating or expressing their pain or emotional state, and may rely on a parent, guardian, or clinical team to detect and monitor either of these two interrelated entities. Insights into pain, emotional health, or overall quality of life are also frequently garnered from a patient's adherence to normal daily activities (eg, going to school).^19^ Although these indirect measures in conjunction with a caretaker's or clinician's account of a patient's pain and overall health are necessary, is important to keep in mind that such assessments come with an added layer of subjectivity and perhaps variability. In the end, multiple factors can prohibit effective and durable pain or psychological interventions from being implemented in young FOP patients, which may impact a patient during adulthood.^20^ What may also be critical for adolescent or adult FOP patients is to not ignore or underreport pain, emotional problems, and other related symptoms^5^ given the benefits of establishing and implementing an early and effective treatment plan.

FOP patients may experience multiple flare‐ups and HO lesions across time and, in some instances, within the same anatomical location.^21^, ^22^, ^23^ Apart from the direct trauma flare‐ups can have on nervous system pain pathways and the peripheral structures they innervate, the occurrence of multiple flare‐ups likely alters the functionality of pain systems both in the periphery and central nervous system (CNS). Taken together, the effect of flare‐up episodes is likely cumulative; potential outcomes of these pathologic events include a facilitation of a chronic pain background or flare‐ups become more painful as they occur in later years of life. This investigation did identify a small subpopulation of FOP patients (*n* = 13) reporting neuropathic pain; however, caution should be used in interpreting this specific finding, because a neuropathic pain phenotype was not identified during a clinical examination or with tools such as the PainDETECT questionnaire.^24^ A more detailed and objective examination of pain and pain phenotypes in FOP is warranted and necessary.

Very little is known regarding the mechanism(s) that drive pain either during or between flare‐up events in FOP, which can contribute to the difficulty in efficiently providing FOP patients with effective analgesia. To date, a variety of analgesic options with mixed efficacy across modalities have been and are commonly employed in FOP.^5^ Glucocorticoids (eg, prednisone), nonsteroidal anti‐inflammatory drugs, COX‐2 inhibitors, and bisphosphonates are frequently prescribed to decrease flare‐ups and the coinciding pain, whereas prescription opioids and gabapentinoids (pregabalin/gabapentin) have little to no analgesic efficacy in patients with FOP.^10^ FOP patients may also refuse or be highly reluctant to consume pharmaceuticals, particularly, opioid‐based therapies, for fear of developing substance use disorder, dependence, or experiencing side effects common to many pharmacological analgesics. Thus, FOP patients and their caretakers may rely and are more receptive to alternative approaches, including cannabinoid oil, cognitive behavioral therapy, Reiki, or mindfulness training. Some individuals find application of heat and cold to be analgesic. In general, the efficacy or lack thereof of various pharmacological and nonpharmacological strategies is derived from individual case reports and systematic evaluations of one or more analgesic approaches have yet to be performed.

The FOP registry has utilized PROMIS, a system developed by the National Institutes of Health that provides a standardized, flexible, and reliable test bank of patient‐reported outcomes for clinical research.^12^, ^25^ Having incorporated comprehensive domains for pain assessment and patient health, PROMIS has been used in many studies to evaluate patients’ pain‐related experience (eg, intensity, interference, quality, and impact), associated physical or mental conditions, and social functions across multiple chronic conditions.^26^, ^27^, ^28^, ^29^, ^30^, ^31^ In order to expand upon findings emanating from the current study, future work may employ the same PROMIS‐based questionnaires in trials evaluating disease‐modifying treatments or therapies aimed at treating FOP symptomology.

A number of novel insights into the dynamics of flare‐up, pain, emotional health, mental health, physical health, and overall quality of life have been gained from analyzing the FOP Registry. Yet the information gathered and presented herein must be considered with the following limitations in mind. First, following the 6‐month follow‐up time point, the total number of FOP patients subsided substantially. For example, whereas 52 total FOP patients with or without flare‐up and in either pain subpopulation were analyzed at 6 months, this number was reduced to 27 at the 30‐month follow‐up. The cause of this decrease is multifactorial and may stem from the recent launch of the registry, patients lost to follow‐up, or incomplete clinical questionnaires, and patient characteristics (eg, willingness of continued participation that may be driven by the factors such as FOP disease or pain severity). Study participants who entered and remained active throughout the 30‐month period were also likely to be on various medications. Variability in medications consumed across patients and time points may have impacted flare‐up, pain, and other health‐related measures. Finally, although flare‐up episodes in some cases are clearly evident, in some circumstances these subcutaneous events may be difficult to detect in an objective manner. Currently, there is an absence of validated methods and clinical biomarkers that can be used to detect and monitor flare‐ups in FOP patients. If such methodologies were available, it would potentially be more feasible and accurate to determine whether and how pain, emotional health, and related health measures track with flare‐up episodes in FOP.

## Disclosures

All authors state that they have no conflicts of interest.

## Supporting information

Supporting Data S1.
