# Supplementary material for: Longitudinal Evaluation of Pain, Flare‐Up, and Emotional Health in Fibrodysplasia Ossificans Progressiva: Analyses of the International FOP Registry
Source: JBMR Plus. 2019 Mar 1;3(8):e10181. doi: 10.1002/jbm4.10181 (PMC6715827; doi:10.1002/jbm4.10181)
Supplement: Supplementary file 1 — Supporting Data S1. [file JBM4-3-na-s001.pdf]

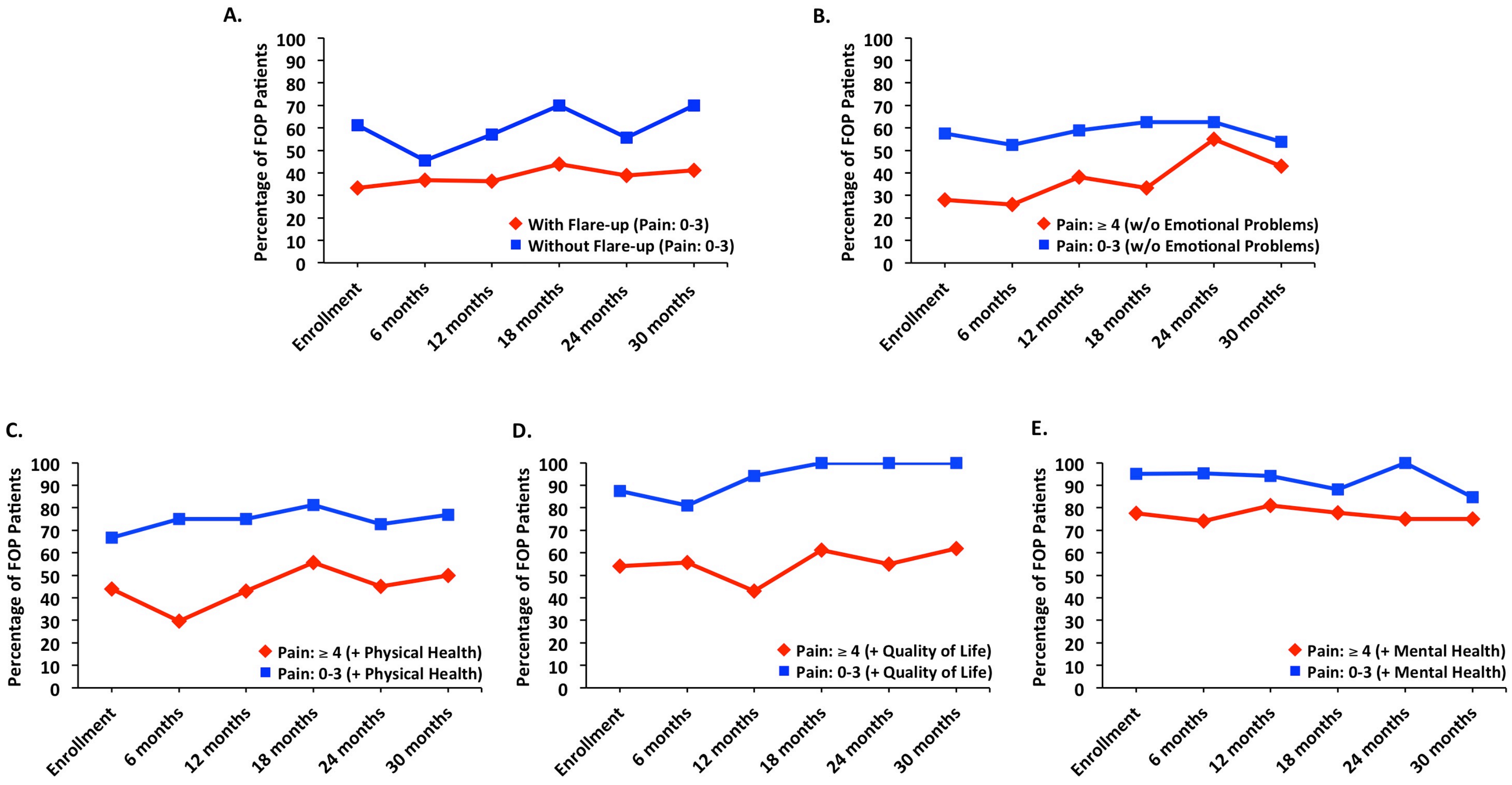

Supplemental Fig. 1: A. the percentage of patients reporting no to mild pain (0-3/10) with and without flare-up; **B-E.** the percentage of patients with different levels of pain reporting no emotional problems (**B**), good physical health (**C**), good qualify of life (**D**) and good mental health **(E**).

Supplemental Table 1: Flare-ups and mental health.

Visit	Response	With Flare-up (N=34) n (%)	Without Flare-up (N=22) n (%)	Total (N=56) n (%)	p-value [1]
In general, how would you rate your mental health, including your mood and your ability to think?					
Enrollment	Total # of Subjects	33	18	51	0.2331
	Class 1: Excellent, Very Good, Good	26 (78.8)	17 (94.4)	43 (84.3)	
	Class 2: Fair, Poor	7 (21.2)	1 (5.6)	8 (15.7)	
Month 6 Follow up	Total # of Subjects	31	22	53	0.1196
	Class 1: Excellent, Very Good, Good	24 (77.4)	21 (95.5)	45 (84.9)	
	Class 2: Fair, Poor	7 (22.6)	1 (4.5)	8 (15.1)	
Month 12 Follow up	Total # of Subjects	22	14	36	0.1336
	Class 1: Excellent, Very Good, Good	17 (77.3)	14 (100.0)	31 (86.1)	
	Class 2: Fair, Poor	5 (22.7)	0 (0.0)	5 (13.9)	
Month 18 Follow up	Total # of Subjects	16	10	26	0.1213
	Class 1: Excellent, Very Good, Good	11 (68.8)	10 (100.0)	21 (80.8)	
	Class 2: Fair, Poor	5 (31.3)	0 (0.0)	5 (19.2)	
Month 24 Follow up	Total # of Subjects	18	9	27	0.2677
	Class 1: Excellent, Very Good, Good	14 (77.8)	9 (100.0)	23 (85.2)	
	Class 2: Fair, Poor	4 (22.2)	0 (0.0)	4 (14.8)	
Month 30 Follow up	Total # of Subjects	16	10	26	0.3524
	Class 1: Excellent, Very Good, Good	11 (68.8)	9 (90.0)	20 (76.9)	
	Class 2: Fair, Poor	5 (31.3)	1 (10.0)	6 (23.1)	

Supplemental Table 2: Flare-ups and emotional health.

Visit	Response	With Flare-up (N=34) n (%)	Without Flare-up (N=22) n (%)	Total (N=56) n (%)	p-value [1]
How often have you been bothered by emotional problems such as feeling anxious, depressed or irritable?					
Enrollment	Total # of Subjects	33	18	51	1.0000
	Class 1: Never, Rarely	16 (48.5)	9 (50.0)	25 (49.0)	
	Class 2: Sometimes, Often, Always	17 (51.5)	9 (50.0)	26 (51.0)	
Month 6 Follow up	Total # of Subjects	31	22	53	1.0000
	Class 1: Never, Rarely	12 (38.7)	8 (36.4)	20 (37.7)	
	Class 2: Sometimes, Often, Always	19 (61.3)	14 (63.6)	33 (62.3)	
Month 12 Follow up	Total # of Subjects	22	14	36	0.5007
	Class 1: Never, Rarely	11 (50.0)	9 (64.3)	20 (55.6)	
	Class 2: Sometimes, Often, Always	11 (50.0)	5 (35.7)	16 (44.4)	
Month 18 Follow up	Total # of Subjects	16	9	25	0.0330
	Class 1: Never, Rarely	6 (37.5)	8 (88.9)	14 (56.0)	
	Class 2: Sometimes, Often, Always	10 (62.5)	1 (11.1)	11 (44.0)	
Month 24 Follow up	Total # of Subjects	18	9	27	0.1032
	Class 1: Never, Rarely	7 (38.9)	7 (77.8)	14 (51.9)	
	Class 2: Sometimes, Often, Always	11 (61.1)	2 (22.2)	13 (48.1)	
Month 30 Follow up	Total # of Subjects	17	10	27	0.4401
	Class 1: Never, Rarely	7 (41.2)	6 (60.0)	13 (48.1)	
	Class 2: Sometimes, Often, Always	10 (58.8)	4 (40.0)	14 (51.9)	

Supplemental Table 3: Anxiety and physical health.

Visit	Response	Without Anxiety (Never, Rarely) (N=37) n (%)	With Anxiety (Sometimes, Often, Always) (N=52) n (%)	Total (N=89) n (%)	p-value [1]
In general, how would you rate your physical health?					
Enrollment	Total # of Subjects	37	52	89	0.0897
	Class 1: Excellent, Very Good, Good	24 (64.9)	24 (46.2)	48 (53.9)	
	Class 2: Fair, Poor	13 (35.1)	28 (53.8)	41 (46.1)	
Month 6 Follow up	Total # of Subjects	24	23	47	0.5639
	Class 1: Excellent, Very Good, Good	13 (54.2)	10 (43.5)	23 (48.9)	
	Class 2: Fair, Poor	11 (45.8)	13 (56.5)	24 (51.1)	
Month 12 Follow up	Total # of Subjects	17	20	37	1.0000
	Class 1: Excellent, Very Good, Good	10 (58.8)	11 (55.0)	21 (56.8)	
	Class 2: Fair, Poor	7 (41.2)	9 (45.0)	16 (43.2)	
Month 18 Follow up	Total # of Subjects	16	18	34	0.4768
	Class 1: Excellent, Very Good, Good	12 (75.0)	11 (61.1)	23 (67.6)	
	Class 2: Fair, Poor	4 (25.0)	7 (38.9)	11 (32.4)	
Month 24 Follow up	Total # of Subjects	12	19	31	1.0000
	Class 1: Excellent, Very Good, Good	7 (58.3)	10 (52.6)	17 (54.8)	
	Class 2: Fair, Poor	5 (41.7)	9 (47.4)	14 (45.2)	
Month 30 Follow up	Total # of Subjects	17	16	33	0.2960
	Class 1: Excellent, Very Good, Good	12 (70.6)	8 (50.0)	20 (60.6)	
	Class 2: Fair, Poor	5 (29.4)	8 (50.0)	13 (39.4)	

Supplemental Table 4: Anxiety and quality of life.

Visit	Response	Without Anxiety (Never, Rarely) (N=37) n (%)	With Anxiety (Sometimes, Often, Always) (N=53) n (%)	Total (N=90) n (%)	p-value [1]
In general, would you say your quality of life is					
Enrollment	Total # of Subjects	37	53	90	0.0119
	Class 1: Excellent, Very Good, Good	31 (83.8)	31 (58.5)	62 (68.9)	
	Class 2: Fair, Poor	6 (16.2)	22 (41.5)	28 (31.1)	
Month 6 Follow up	Total # of Subjects	24	24	48	0.1246
	Class 1: Excellent, Very Good, Good	19 (79.2)	13 (54.2)	32 (66.7)	
	Class 2: Fair, Poor	5 (20.8)	11 (45.8)	16 (33.3)	
Month 12 Follow up	Total # of Subjects	17	21	38	0.3068
	Class 1: Excellent, Very Good, Good	13 (76.5)	12 (57.1)	25 (65.8)	
	Class 2: Fair, Poor	4 (23.5)	9 (42.9)	13 (34.2)	
Month 18 Follow up	Total # of Subjects	16	19	35	0.0964
	Class 1: Excellent, Very Good, Good	15 (93.8)	13 (68.4)	28 (80.0)	
	Class 2: Fair, Poor	1 (6.3)	6 (31.6)	7 (20.0)	
Month 24 Follow up	Total # of Subjects	12	19	31	1.0000
	Class 1: Excellent, Very Good, Good	9 (75.0)	13 (68.4)	22 (71.0)	
	Class 2: Fair, Poor	3 (25.0)	6 (31.6)	9 (29.0)	
Month 30 Follow up	Total # of Subjects	17	16	33	0.4384
	Class 1: Excellent, Very Good, Good	14 (82.4)	11 (68.8)	25 (75.8)	
	Class 2: Fair, Poor	3 (17.6)	5 (31.3)	8 (24.2)	

Supplemental Table 5: Sleep and emotional health.

Visit	Response	With Sleeping Problem (N=36) n (%)	Without Sleeping Problem (N=57) n (%)	Total (N=93) n (%)	p-value [1]
How often have you been bothered by emotional problems such as feeling anxious, depressed or irritable?					
Enrollment	Total # of Subjects	35	55	90	0.0784
	Class 1: Never, Rarely	10 (28.6)	27 (49.1)	37 (41.1)	
	Class 2: Sometimes, Often, Always	25 (71.4)	28 (50.9)	53 (58.9)	
Month 6 Follow up	Total # of Subjects	17	33	50	0.2184
	Class 1: Never, Rarely	4 (23.5)	15 (45.5)	19 (38.0)	
	Class 2: Sometimes, Often, Always	13 (76.5)	18 (54.5)	31 (62.0)	
Month 12 Follow up	Total # of Subjects	16	23	39	0.7475
	Class 1: Never, Rarely	7 (43.8)	12 (52.2)	19 (48.7)	
	Class 2: Sometimes, Often, Always	9 (56.3)	11 (47.8)	20 (51.3)	
Month 18 Follow up	Total # of Subjects	17	17	34	0.7319
	Class 1: Never, Rarely	7 (41.2)	9 (52.9)	16 (47.1)	
	Class 2: Sometimes, Often, Always	10 (58.8)	8 (47.1)	18 (52.9)	
Month 24 Follow up	Total # of Subjects	13	18	31	0.2936
	Class 1: Never, Rarely	6 (46.2)	12 (66.7)	18 (58.1)	
	Class 2: Sometimes, Often, Always	7 (53.8)	6 (33.3)	13 (41.9)	
Month 30 Follow up	Total # of Subjects	14	21	35	0.1662
	Class 1: Never, Rarely	4 (28.6)	12 (57.1)	16 (45.7)	
	Class 2: Sometimes, Often, Always	10 (71.4)	9 (42.9)	19 (54.3)	

Supplemental Table 6: Sleep on mental health.

Visit	Response	With Sleeping Problem (N=36) n (%)	Without Sleeping Problem (N=58) n (%)	Total (N=94) n (%)	p-value [1]
In general, how would you rate your mental health, including your mood and your ability to think?					
Enrollment	Total # of Subjects	35	55	90	0.0282
	Class 1: Excellent, Very Good, Good	26 (74.3)	51 (92.7)	77 (85.6)	
	Class 2: Fair, Poor	9 (25.7)	4 (7.3)	13 (14.4)	
Month 6 Follow up	Total # of Subjects	17	33	50	0.1022
	Class 1: Excellent, Very Good, Good	12 (70.6)	30 (90.9)	42 (84.0)	
	Class 2: Fair, Poor	5 (29.4)	3 (9.1)	8 (16.0)	
Month 12 Follow up	Total # of Subjects	16	23	39	0.1387
	Class 1: Excellent, Very Good, Good	12 (75.0)	22 (95.7)	34 (87.2)	
	Class 2: Fair, Poor	4 (25.0)	1 (4.3)	5 (12.8)	
Month 18 Follow up	Total # of Subjects	18	17	35	1.0000
	Class 1: Excellent, Very Good, Good	15 (83.3)	14 (82.4)	29 (82.9)	
	Class 2: Fair, Poor	3 (16.7)	3 (17.6)	6 (17.1)	
Month 24 Follow up	Total # of Subjects	13	18	31	1.0000
	Class 1: Excellent, Very Good, Good	11 (84.6)	15 (83.3)	26 (83.9)	
	Class 2: Fair, Poor	2 (15.4)	3 (16.7)	5 (16.1)	
Month 30 Follow up	Total # of Subjects	14	20	34	0.0423
	Class 1: Excellent, Very Good, Good	8 (57.1)	18 (90.0)	26 (76.5)	
	Class 2: Fair, Poor	6 (42.9)	2 (10.0)	8 (23.5)	
